# Can artificial intelligence reach human thought?

**DOI:** 10.1093/pnasnexus/pgad409

**Published:** 2023-12-19

**Authors:** Athanassios S Fokas

**Affiliations:** Department of Applied Mathematics and Theoretical Physics, University of Cambridge, Cambridge CB3 0WA, UK; Viterbi School of Engineering, University of Southern California, Los Angeles, CA 90089, USA; Mathematics Research Center, Academy of Athens, Athens 11527, Greece

**Keywords:** Gödel’s theorem and AI, body proper and AI, glia cells and AI, unconscious process and AI, creativity versus AI

## Abstract

The transformative achievements of deep learning have led several scholars to raise the question of whether artificial intelligence (AI) can reach and then surpass the level of human thought. Here, after addressing methodological problems regarding the possible answer to this question, it is argued that the definition of intelligence proposed by proponents of the AI as “the ability to accomplish complex goals,” is appropriate for machines but does not capture the essence of human thought. After discussing the differences regarding understanding between machines and the brain, as well as the importance of subjective experiences, it is emphasized that most proponents of the eventual superiority of AI ignore the importance of the *body proper* on the brain, the *laterization* of the brain, and the vital role of the glia cells. By appealing to the incompleteness theorem of Gödel’s and to the analogous result of Turing regarding computations, it is noted that consciousness is much richer than both mathematics and computations. Finally, and perhaps most importantly, it is stressed that artificial algorithms attempt to mimic only the conscious function of parts of the cerebral cortex, ignoring the fact that, not only every conscious experience is preceded by an unconscious process but also that the passage from the unconscious to consciousness is accompanied by loss of information.

## Introduction

The process of combining ingenious mathematical algorithms with extremely powerful computers allows scientists and engineers to focus with great precision on particular aspects of reality and to attain highly complex goals. The apogee of this process is reached with artificial intelligence (AI), which provides the next step in the hierarchy of great human endeavors that began with writing and printing and continued with computing and the internet.

Progress in AI and, in particular, the emergence of deep learning, coupled with the availability of powerful computers, have led to the surprising development that machines have defeated humans in a variety of games, starting with the triumph of IBM’s Deep Blue, which in 1997 overpowered the chess champion Garry Kasparov.^a^ The achievements of AI in chess, Go, and video games have been extensively publicized and, naturally, have created wide interest. However, the main reason for the excitement generated by AI is its success not in games but in a variety of real-life situations, which include the following: automatization of routine labor tasks, understanding of speech and images, and mechanization of certain medical diagnoses. There are many applications from voice, speech, text recognition and translation of a large number of languages, to protein folding, development of new antibiotics, and driverless cars.

The far-reaching achievements of deep learning, such as ChatGPT, have led several scholars to raise the question of whether AI can reach the level of “artificial general intelligence,” namely, whether AI can reach and then surpass the level of human thought. The milestone where AI, supposing, reaches human intelligence was called by Vernor Vinge, the “singularity.” This notion has been popularized by the futurist scholar and director of Google Engineering, Ray Kurzweil (1).

There is in my opinion a serious *methodological problem* concerning the possible proof that a machine has surpassed the human level of intelligence. This would require a proof that for *every* conceivable human goal, the machine achieves better performance. So far, this has been accomplished for *particular* goals by the direct competition of a human expert and a machine. For example, this happened for the goals of winning a chess or a Go game. However, it is apparent that such an approach cannot be used for an uncountable number of possible situations. Hence, unless a different methodology is suggested, the question of proving whether general AI has been reached is not well defined.

In any case, even if we assume that this question is well-posed, its analysis, necessitates, first, the introduction of a definition of intelligence. The cosmologist and leading AI exponent, Max Tegmark, attempted to provide such a definition. He defined intelligence as “the ability to accomplish complex goals.” In his important book, *Life 3.0: being human in the age of artificial intelligence* (2), it is claimed that this definition encompasses Oxford Dictionary’s definition of intelligence, “as the ability to acquire and apply knowledge and skills,” as well as several of the definitions proposed in the *Nobel Week Dialogue 2015*: *The Future of Intelligence*. In this conference, among the definitions proposed for intelligence were, “the capacity for problem solving, learning, logic, and planning.” According to Tegmark, acquiring knowledge, learning new information or a new skill, solving specific problems, employing logical algorithms, and designing concrete plans, can all be considered as processes subsumed by the phrase “accomplishing complex goals.”

It will be argued below, that although Tegmark’s definition of intelligence is adequate for machines, it does not capture the essence of human thought. Indeed, I believe that this definition is appropriate for technology, which can be defined as

a collection of devices and engineering practices as means of achieving a complicated goal.

## Understanding

Even if for the sake of argument, we assume that Tegmark’s definition is adequate, it is important to note that human *understanding* differs from that of machines, and this has serious implications on the *types of goals* that machines can be superior to humans. For instance, in machine translation, the computer, after being trained with massive data, discovers complex relations among words, from which it can defer other relations. For example, the computer can “understand” that the pair “king” and “queen” has analogies with the pair “husband” and “wife.” However, the type of relations established by the computer is of a completely different nature to the relations established via human associations whose crucial importance in our thought has been emphasized in my book, *Ways of Comprehending* (3).

Conscious and unconscious associations establish relations on the basis of deep understanding of the underlying constituent parts, whereas the computer does not understand the meaning of these parts.

As the computer scientist and Turing prize winner, Joseph Sifakis, points out, the relations obtained via AI *give rise to predictability*, but in contrast to the scientific knowledge generated by the brain, they *do not yield understanding of the underlying processes* (4). For example, regarding language, the computer forms syntactic as opposed to semantic relations. For this reason, it is not surprising that AI still performs poorly in the so-called “Winograd Schema Challenge” (5). This is exemplified by the challenge to determine what the word “they” refers to in the following two sentences: “The city councilmen refused the demonstrators a permit because they feared violence,” and “The city councilmen refused the demonstrators a permit because they advocate violence.” This raises questions of whether a machine can pass a carefully designed “Turing test,” namely, whether a machine can converse in writing well enough to trick a person into thinking that is conversing with another human.

## Implementation

The process of *implementing* goals is obviously drastically different between machines and humans.

The embodied brain shares with artificial circuits the ability to store and process information, but in addition, the brain creates subjective experiences. An individual, while achieving a particular goal feels in a unique way an embodied fulfillment.

This fundamental difference was perfectly captured by Kasparov who, describing his defeat by the IBM’s artificial creation, wrote that at least the Deep Blue “was not enjoying beating me.” The elements of “emotional intelligence” and of “self-awareness” suggested in the definition of intelligence in the 2015 meeting mentioned earlier, are *not* contained in the definition of intelligence as “accomplishing complex goals.”^b^

In this regard, it should not be ignored that human’s underlying emotional intelligence originates from primordial desires related to the vital evolutionary goals of self-preservation (avoiding death) and reproduction (enjoying sex). This means that this type of intelligence is extremely broad and important (6). In addition, organisms have the ability to be informed *directly* from their environment, as opposed of needing to be programmed by an external agent in order to be able to receive a particular type of information.

## Creativity

The above remarks summarize, in a sense self-evident differences between artificial and human intelligence. Beyond these differences, the suggestion that human thought can be simply reduced to the property of “accomplishing complex goals” is, in my opinion, fundamentally wrong. This becomes clear by looking at a most important component of intelligence, namely, creativity, which, incidentally, was included in the definitions proposed in the 2015 conference. I believe that,

creativity is not defined in terms of achieving a specific goal. On the contrary, it is measured by the distance of the final unexpected achievement from a starting vague idea. Indeed, the defining property of creative individuals is their capability of establishing far remote associations and generating completely unexpected relations between different topics. These topics had appeared until that point so distinct, that no one had thought of posing the goal of establishing a connection between them. As stated repeatedly in (3), the process of establishing such remote associations is mostly unconscious and therefore much more difficult to be “programmed.” In this sense, it can be claimed that the origin of human creativity is nonliteral and nonalgorithmic, rather, it is largely metaphorical, imaginative, and transcendental.

Einstein expressed clearly the nonliteral nature of creativity when he stated that, “The words or the language as written or spoken do not seem to play any role in the mechanism of my thought” (7, p. 32).

Regarding human creativity, it seems to me that,

the less the final achievement is predefined and the more it is free from preconditions, the less the creative process is affected from misconceptions and the current way of thinking about a given problem. Hence, the higher the chances for a breakthrough.

For example, the creation by Picasso of Olga in an armchair (1917) was the result of the great master’s specific goal to depict Olga Koklova, who soon afterwards became his first wife. But what was the goal associated with *Guernica* (1937)? Picasso’s vague motivation of depicting the atrocities of the Spanish civil war, gave rise to this specific artistic creation that arose directly from Picasso’s unconscious. By comparing these two works, it becomes clear that in Arts, the less a goal is a priori defined, the higher the value of the artistic creation. The same is true in Mathematics and the Sciences.

Regarding Mathematics, in what follows I will discuss two examples from my own work. The imaging technique of electroencephalography (EEG) is based on the fact that a specific mental process is associated with brain activation of a unique form, which expresses itself via the generation of a specific neuronal *electric current*. This current gives rise to an electric potential which can be measured on the scalp. EEG gives rise to the important “inverse mathematical problem” of computing the neuronal current from the knowledge of the measured electric potential. A general algorithm for determining the current was introduced in (8). The numerical implementation of this algorithm requires the computation of a certain auxiliary function (9, 10). This well-defined goal can be achieved via the training of a two-layer neural network, which provides yet another illustration of the importance of machine learning (11). The solution of this specific goal is conceptually very different to my work on the Lindelöf hypothesis (12), where a novel approach was introduced to this historical problem.^c^ The key step of this approach is the derivation of a new identity satisfied by the Riemann zeta function. The genesis of this unexpected identity was not the result of a specific goal, but the outcome of unconscious processes motivated by my vague idea of imbedding the Riemann zeta function in a larger mathematical framework.

Regarding the Sciences and in particular Physics, Richard Feynman had expressed clearly the fact that breakthroughs are not the result of well-defined goals. He wrote: “Whatever way comes out, it’s nature, and she is going to come out the way she is! Therefore, when we go to investigate it, we shouldn’t predecide what it is we’re going to find.”

## From platonism to AI

AI provides the apogee of mathematics, of algorithms, and in general of the rational. In this sense, it represents the strongest possible endorsement of key elements of Platonism. As noted in the epilog of Fokas (3), the overreliance of Plato on reason led him to elevate theoretical constructions, and in particular his *Forms*, *above* reality. For example, according to Plato, the stars are less important than their orbits. He argued that stars can be observed directly, so they are susceptible to the misleading information gathered by the “unreliable senses”, whereas their orbits can be inferred via the “perfection of theoretical reasoning”. He wrote that, “the stars […] are far inferior […] to the orbits that carry them, which are perceptible to reason and thought […]” (13). Plato, pushing the importance of the rational to its ultimate limit, reached the erroneous conclusion that the only “true reality” consists of certain rational constructions. Indeed, according to him, his disembodied Forms are the “essence of things,” whereas sensory experiences are simply “shadows of reality.”

In the same way that Plato elevated certain abstract notions above reality, some scholars have elevated *reductionism* and *computability* above the embodied brain and the astounding processes associated with it. These scholars claim that any phenomenon can be fully understood by its reduction to appropriate constituent elements, which can then be simulated via powerful computers. For example, the MIT expert on robotics Rodney Brooks, after presenting the 1955 quote of the pioneer AI innovator, John McCarthy, regarding the “conjecture that every aspect of learning or any other feature of intelligence can in principle be so precisely described that a machine can be made to simulate it,” he states that, “As a materialist reductionist, I agree with that” (14). However, contradicting this claim, he later admits that the current computational approaches are inadequate. Actually, the entire article of Brooks is about possible ways to overcome the limitations of AI. For example, Brooks states: “if we are pushing things into the information metaphor, are we missing things”? And after reviewing certain behavioral experiments involving flatworms, he notes that neurons, in contrast to current AI computations, are adaptive. He writes, “it’s some sort of adaptation and our computation is not locally adaptive.” Later, after correctly noting that the discoveries related to computations and to calculus were in a sense natural, in contrast to the extraordinary “conceptual jumps” needed for quantum mechanics and general relativity, he asks: “The question is whether there is something like that out there that could potentially give us a better [information] metaphor.”

In this connection, it is worth noting that according to evolutionary biology, *intentionality* begins at the *local* level, which means that every individual eukaryotic cell has the capacity to respond to messages from its environment and to adapt accordingly, so that it can survive. The *global* manifestation of this capacity was eloquently expressed by the great philosopher and first preneuroscientist, Baruch Spinosa, with the concept of *conatus*, which is the tendency of life-organisms to maintain their existence. Intentionality is facilitated by *adaptation*, which provides yet another fundamental difference with respect to mechanical systems which cannot adapt.

It appears that most proponents of the superiority of the AI in comparison to the brain, are biased by their knowledge of physical process. In this regard, it must be emphasized that *physics is invariant and predictive*. This is the result of the *existence of fundamental physical laws*. Mathematics is crucial both for formulating and for analyzing these laws. In contrast to physical theories, the theory of biological evolution, merely provides a useful *framework* for understanding biology and certainly it is nonpredictive. *The modus operandi of biological evolution appears to be “trial and error.” Moreover, new solutions are usually sought within the constraints of what already has been constructed.* This explains the abundance in biology of *redundancy*, namely, of the fact that the same task can be accomplished, with varying degree of efficiency, via completely different mechanisms. Evolution invents new improvements, which are used within the existing framework, without necessarily abandoning the use of older inventions. *This gives rise to overlapping systems of high redundancy and immense, “illogical,” complexity*. An example of this process is provided by the plethora of completely different mechanisms used for neuronal plasticity and employed for learning, discussed in (3). This crucial feature of biological evolution, in addition to arguing against the position that organisms are the result of a “grand design,” presumably eliminates the possibility that fundamental biological processes follow *global fundamental laws*. Since mathematics reveals its power precisely in relation to such laws, these arguments suggest that, perhaps mathematics in particular and algorithms in general, cannot have the pivotal impact in biology that they have had in physics. This suggest that, in our effort to achieve deep insight, we should perhaps abandon the dogmatic apotheosis of reductionism and computability, and adopt a more flexible framework. Such a framework was introduced in (3) and is based on notions which reflect basic neuronal mechanisms, including the following: *associations, continuity, generalization, abstraction, plasticity, interconnectedness*, and the dialectic pairs of *local* versus *global processes*, *simplicity* versus *complexity*, and *unification* versus *reduction*.

Incidentally, the trial and error modus operandi of biology is consistent with the “bag-of-tricks hypothesis,” which claims that biological organisms have evolved a specialized set of optimal algorithms for solving particular problems. Anthony Zador has claimed that this highly specialized nature of biological algorithms explains their great efficiency in comparison to algorithms of AI, which, at least until recently, are designed to solve a wide range of problems (15).^d^

## The embodied brain, laterization, and the role of the glia

The following three important elements are usually overlooked by exponents of AI. First, the impact of the body proper on the brain, second, the laterization of the brain, and third, the vital role of the glia cells.

Many of the unconscious processes begin in the body proper as opposed to the brain. Indeed, the brain’s basic functions are hugely influenced by its obvious topological attribute, namely that it is embodied.

In particular, the brain, in addition to the global connectivity exemplified by the thalamocortical system, as well as to the parallel-unidirectional connectivity found in the cerebellum, basal ganglia, and the hippocampus, also possesses a third type of topological connectivity. This consists of a diffused, highly complex set of connections resembling a large multicomponent fan. These connections begin in a variety of *nuclei*, namely in a collection of specialized neurons, located in the brainstem and the hypothalamus. The names of these nuclei are related to the substances they release, and they include noradrenergic, serotonergic, dopaminergic, cholinergic, and histaminergic nuclei. Neurons from these nuclei diffuse to large parts of the brain, and in this way, they influence billions of synapses. Moreover, there exist a large number of molecules, named *neuromodulators* and *hormones*, which play a crucial role in ensuring proper interactions between the body proper and the brain. The impact of the body proper on the brain is exemplified by the fact that 95% of the important neurotransmitter serotonin (which is enhanced by many antidepressant medications) is produced in enteric neurons (16, p. 136).

The brain exhibits *laterization*, namely, the functions of its two hemispheres are not identical. There is an anatomical asymmetry between the two hemispheres which implies a functional asymmetry. Space considerations prevent me from elaborating on the importance of functional asymmetry. It is sufficient to state, that, as clearly illustrated by experiments performed at Caltech in “split brain patients,” this asymmetry is of crucial importance for the proper functioning of the brain (17).

Regarding the role of the glia cells, it is noted that many approaches to AI attempt to mimic functions of the neurons. However, 85% of the brain’s cells are glia cells! The plethora of these cells, together with Aristotle’s aphorism that “Nature does not create anything useless,” suggest that these cells are very important. Indeed, as shown in detail in the excellent book, *The other brain* (18), glia’s role is far more encompassing than the one designated as merely “supportive.” In particular, glia cells excrete a variety of vital substances, and importantly, affect decisively neuronal networks via two different ways: they communicate directly using “gap-junctions” and indirectly via the spread of calcium waves. The latter communication is slow (of the order of seconds), in contrast to the fast (of the order of milliseconds) electrical communication employed by neurons. The slow, steady, nonspecific, global communication of glia, complements and enhances the fast, specific, and local synaptic neuronal communication. This provides yet another mechanism for plasticity, which is crucial for learning and memory.

How can AI expect to reach human thought if it ignores embodiment, laterization and the huge importance of the glia cells? Incidentally, mathematical modeling of the brain’s functions also ignores these fundamental elements.

A decisive argument in favor of the superiority of the human though in comparison with AI is provided by the fact that consciousness is much richer than both mathematics and computations. As argued in (3), this is a direct consequence of Gödel’s *incompleteness theorem*, which states that there exist infinitely many true statements which cannot be formally verified via mathematics (whereas some of these statements are intuitively obvious), and by the analogous result of Turing, which shows that this very serious limitation of mathematics cannot be overcome via mechanical computations. Finally, and perhaps most importantly, artificial algorithms attempt to mimic only the conscious function of parts of the cerebral cortex, ignoring the fact that not only every conscious experience is preceded by an unconscious process but also the passage from the unconscious to consciousness is accompanied by loss of information. This latter fact has been demonstrated by several neuroscientists, and in particular, in the transformative work of Nikos Logothetis on binocular rivalry (19).

The human thought, in contrast to artificial algorithms, is created via the interaction of conscious and unconscious processes in the dynamic environment of the embodied brain, and is crucially affected by hormones, neuromodulators, and a variety of unconscious homeostatic mechanisms.

This implies that human thought is much broader than Mathematics, the Sciences, and Technology, and therefore I do not expect that it can be replaced by AI. This point of view is consistent with positions expressed by several other scholars, see for example (20) and (21).

Interestingly, machine learning provides a clear illustration of the ingenuity of Nature. Indeed, a child develops various skills, including linguistic ability and the cognitive capacity to manipulate complicated concepts, not in an analytic way by following specific logical rules and algorithms, but in a holistic manner via the process of imitation. Deep learning is successful precisely because it is closer to the way that the brain learns than the logic-based earlier forms of AI. Similarly, the success of “deep reinforcement learning” is consistent with the fact that it mimics the important psychological process of positive reinforcement.

The above analysis is based on the current advances of AI. It would certainly require extensive modifications if a new approach is developed based on a hybrid, silicon–neuron chip.

The more evident becomes the transformative impact of AI to our way of living, the more imperative it is for scientists and engineers involved with AI to resist the ongoing hype and to remain humble. A mature researcher, although aware that the nature of highly complex physical and man-made phenomena (such as how the computer “understands” important relations via the analysis of “big data”) can never be fully knowledgeable, continues to live a life dedicated to searching. This will inevitably lead to further progress and occasionally to breakthroughs. In this way, they will again feel, not only deep eudemonia but also awe for the inventiveness of Nature as expressed in its greatest creation, which remains the human brain.

## Notes

a This algorithm was not based on deep learning, despite the use of the word “Deep.”

b Surprising, although it is stated in (2, p. 184) that “If we can one day figure out what properties an information-processing system needs in order to have a subjective experience […],” in page 50 of the same book, the contradictory claim is made that emotional intelligence and self-awareness are contained in the definition of “accomplishing complex goals.”

c Lindelöf’s hypothesis is closely related with the Riemann hypothesis which is the most famous open problem in the history of mathematics.

d Another difference with physics is that biology does not follow the model of the “scientific revolutions,” described by Thomas Kuhn. This was emphasized by the leading evolutionary biologist Ernst Mayr (1904–2005) in *The Growth of Biological Thought* (22).
